# Acute arterial occlusive disease due to tumor thrombus from lung metastasis of breast cancer with cartilaginous and osseous metaplasia: a case report

**DOI:** 10.1186/s40792-023-01598-8

**Published:** 2023-02-01

**Authors:** Ayana Sato, Akiko Matsumoto, Saki Naruse, Yuka Isono, Yuka Maeda, Miki Yamada, Tatsuhiko Ikeda, Yuko Sasajima, Hiromitsu Jinno

**Affiliations:** 1 Department of Surgery, Teikyo University School of Medicine, 2-11-1 Kaga, Itabashi, Tokyo, 173-8606 Japan; 2 Department of Pathology, Teikyo University School of Medicine, 2-11-1 Kaga, Itabashi, Tokyo, 173-8606 Japan

**Keywords:** Breast cancer, Cartilaginous and/or osseous metaplasia, Lung metastases, Tumor embolism

## Abstract

**Background:**

Tumor embolization due to venous infiltration of breast cancer pulmonary metastases is very rare.

**Case presentation:**

A 72-year-old female was diagnosed with triple-negative breast cancer. Neoadjuvant chemotherapy was discontinued because of progressive disease, and a right mastectomy with sentinel lymph node biopsy was performed. The pathological analysis of surgical specimens revealed carcinoma with cartilaginous and/or osseous metaplasia. At 22 months after surgery, lung metastasis was observed, and 6 months after initiating treatment for lung metastases, she complained of sudden numbness in the left-lower limb with trouble walking. Ultrasonography showed an embolism in the left popliteal artery, and contrast computed tomography showed enlarged lung metastases and infiltration of the left-upper lobe disease into the left superior pulmonary vein and left atrium. Acute arterial occlusive disease in the left-lower limb caused by the tumor embolism was suspected, so an endovascular thrombectomy was performed. Tumor emboli were removed by embolectomy catheter.

**Conclusion:**

This report of lung metastasis from breast cancer with cartilaginous and/or osseous metaplasia and acute lower-limb artery occlusion due to a tumor thrombus adds useful information to the literature on these extremely rare cases.

## Background

The incidence of breast cancer with cartilaginous and/or osseous metaplasia ranges from 0.003 to 0.12% of invasive ductal carcinomas. We report an extremely rare case of lung metastasis from breast cancer with cartilaginous and/or osseous metaplasia and acute lower-limb artery occlusion due to a tumor thrombus.

## Case presentation

**Fig. 1:**
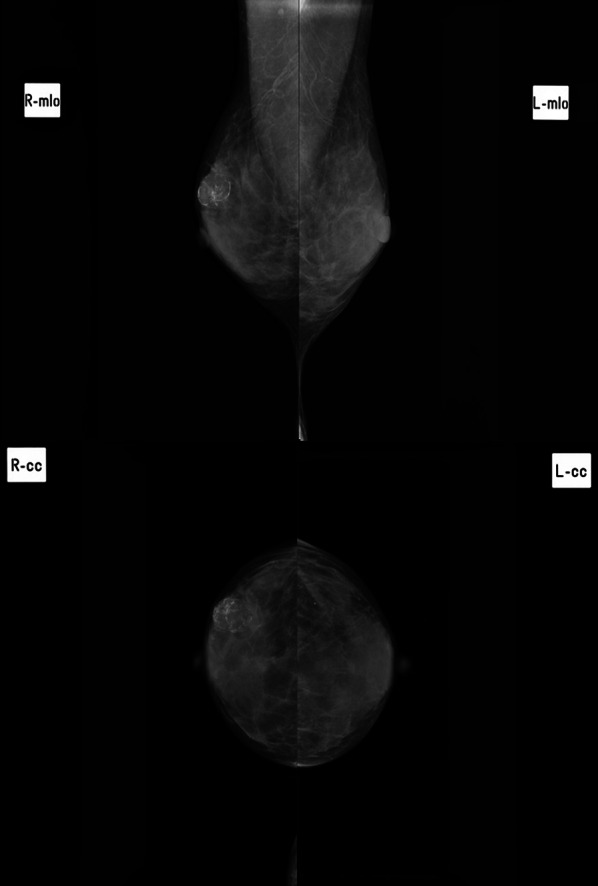
Mammography showing a focal asymmetric density with a calcified lesion at the middle-outer portion of the right breast

**Fig. 2:**
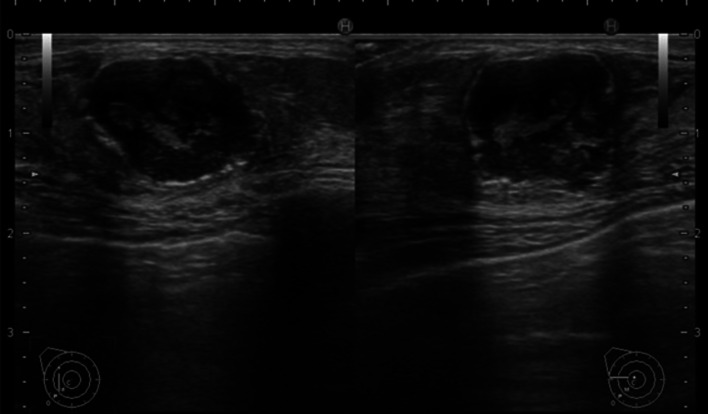
Ultrasonograph showing a 1.8-cm hypoechoic irregular mass at the 9 o’clock position of the right breast

**Fig. 3:**
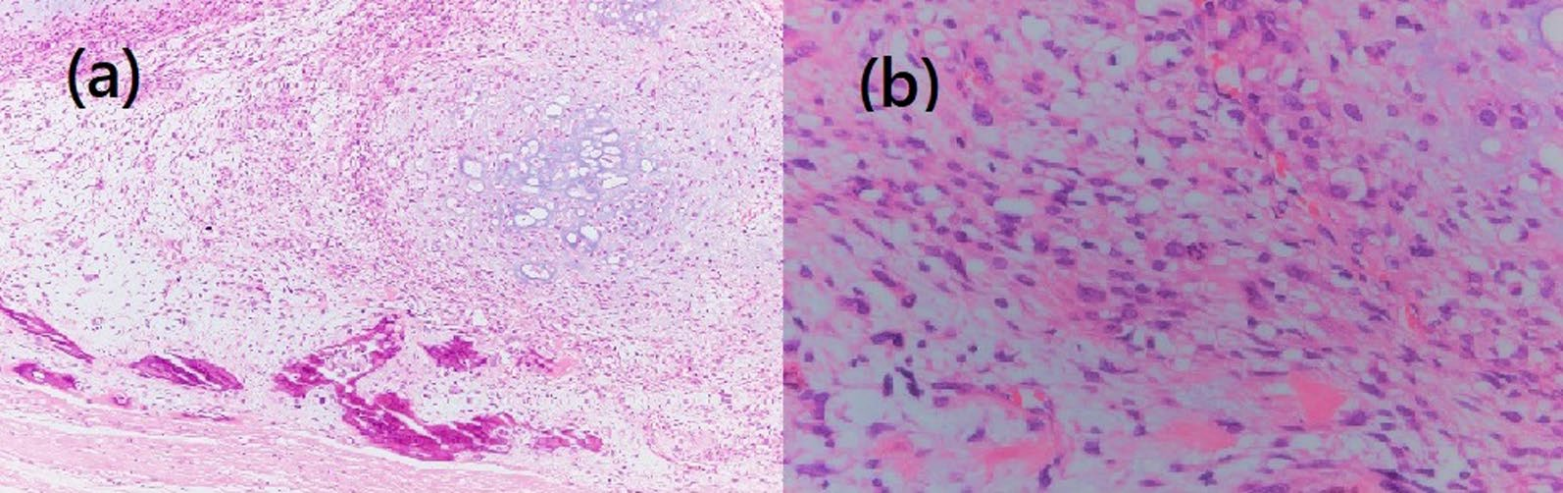
Histological findings (hematoxylin and eosin staining). Tumor cells are scattered in the myxoid matrix with cartilage differentiation. Ossification is seen at the tumor margin (**a** × 4). Tumor cells have large and irregular nuclei with hyperchromasia (**b** × 40)

**Fig. 4:**
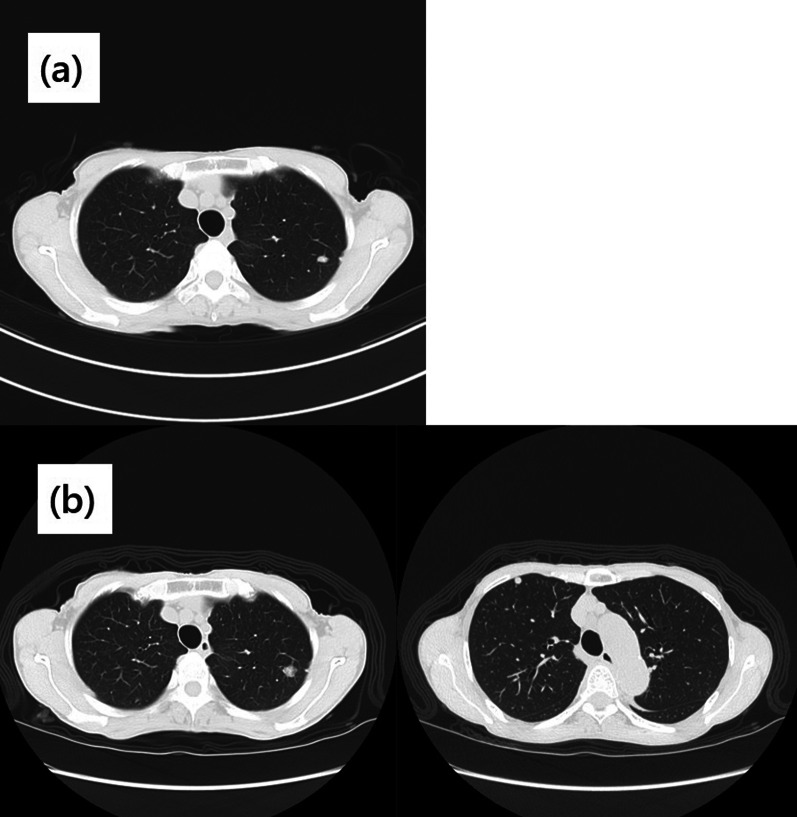
Computed tomography showing a pulmonary nodule. **a** 22 months after surgery; **b** 25 months after surgery

A 72-year-old female patient was referred for evaluation of a right breast mass. Physical examination showed a 2-cm hard mass at the 9 o’clock position of the right breast. She had no remarkable medical history, but her family history included an older sister and maternal aunt who had breast cancer. Mammography revealed a focal asymmetric density with a calcified lesion at the middle-outer portion of the right breast (Fig. 1). Ultrasonography (US) showed a 1.8-cm hypoechoic irregular mass at the 9 o’clock position of the right breast (Fig. 2), with no lymphadenopathy in the axilla. Histopathological examination of tissue samples acquired by performing a US-guided core-needle biopsy of the tumor showed matrix-producing carcinoma that was estrogen receptor (ER)-negative, progesterone receptor (PgR)-negative, and human epidermal growth factor receptor 2 (HER2)-negative immunohistochemically. Positron-emission tomography with computed tomography (CT) revealed neither distant nor lymph node metastasis. Neoadjuvant chemotherapy (NAC) with four cycles of nab-paclitaxel (260 mg/m^2^) followed by four cycles of CEF (cyclophosphamide 500 mg/m^2^, epirubicin 100 mg/m^2^, and fluorouracil 500 mg/m^2^) was initially planned. After the first cycle of nab-paclitaxel, the original tumor had enlarged to 2.7 cm, and a new lesion was present. Although NAC was switched to CEF therapy, the breast disease was determined to be progressive because of the 72% increase in the main tumor size after only one cycle of CEF. Three weeks after the CEF administration, a right mastectomy with sentinel lymph node biopsy was performed. The pathological analysis of the surgical specimens revealed a carcinoma with cartilaginous and/or osseous metaplasia (Fig. 3), pathological invasive size of 2.1 cm, and no sentinel lymph node metastasis (0/2). Immunohistochemical examination showed a triple-negative type tumor with a ki67 expression level of 20%. After surgery, the patient received post-mastectomy radiation and adjuvant chemotherapy with eight courses of capecitabine (1500 mg/m^2^/day, 2 weeks on followed by 1 week off in a 3-week cycle for each course). Twenty-two months after surgery, CT showed a pulmonary nodule approximately 0.9 cm in diameter in the left-upper lobe. Because it was a single nodule, watchful waiting was chosen. After 3 months, the nodule had enlarged to 1.1 cm, and a new nodule in the right-upper lobe was observed (Fig. 4); thus, video-assisted thoracoscopic partial resection of the right lung nodule was performed. Histopathological examination showed metastasis of the triple-negative breast carcinoma with cartilaginous and osseous metaplasia. A BRCA1/2 genetic examination was subsequently performed, and no mutation was found.

**Fig. 5:**
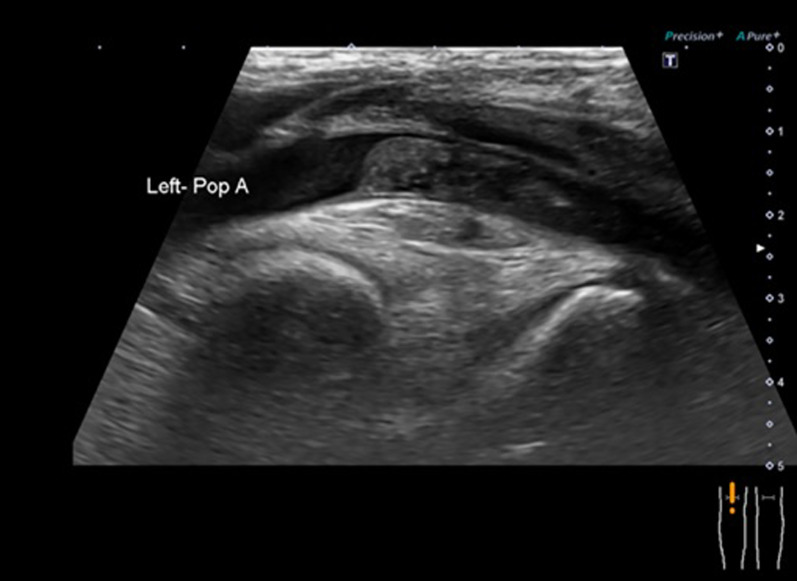
Ultrasonograph showing an embolism in the left popliteal artery

**Fig. 6:**
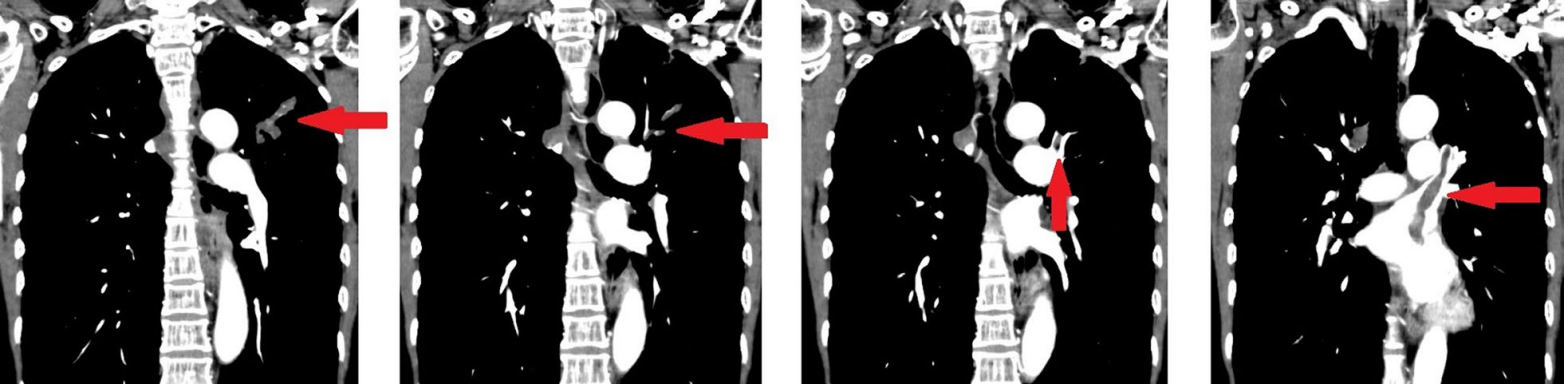
Contrast computed tomography showing enlarged lung metastases and that disease in the left-upper lobe has infiltrated the left superior pulmonary vein and left atrium

**Fig. 7:**
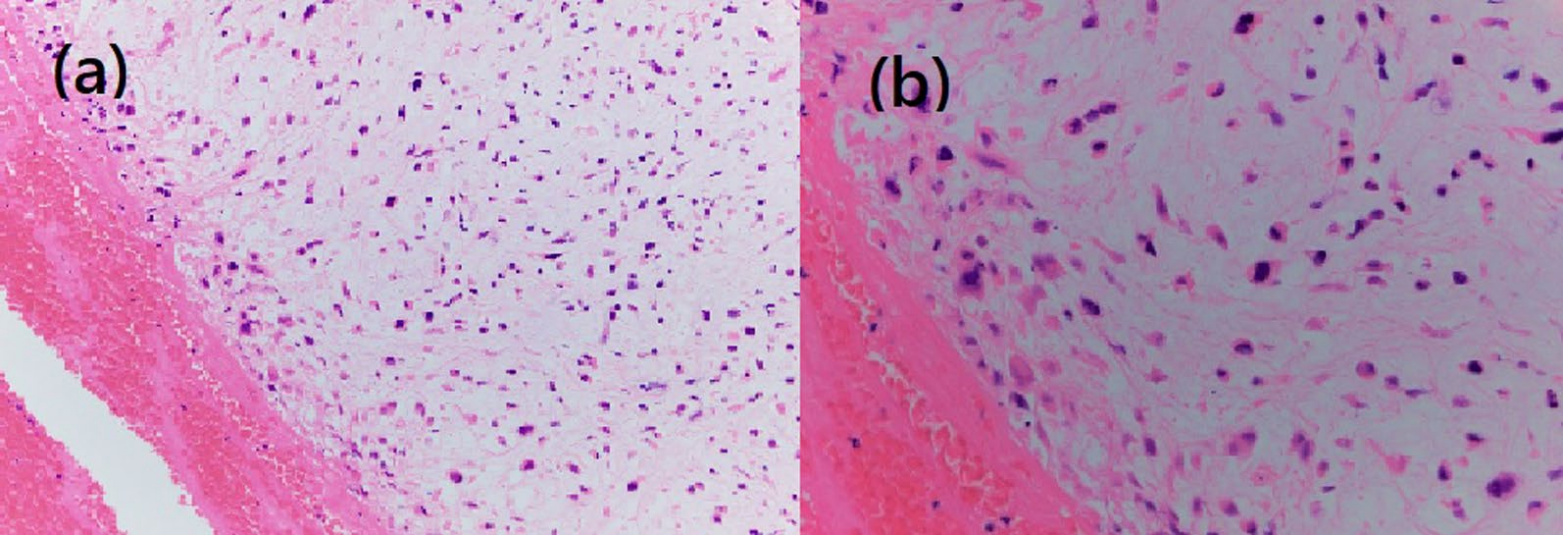
Atypical spindle cells are suspended in a chondromyxoid matrix. Tumor cells show hyperchromatic pleomorphic nuclei. **a** × 20; **b** × 40

Although S-1 (100 mg/day) was started as first-line therapy for recurrent disease, the left-lung nodule had enlarged to 2.3 cm, and new lesions had appeared in the left lung. Treatment was switched to eribulin (1.4 mg/m^2^) after completion of three cycles of S-1, and after the second cycle, she complained of a sudden numbness in her left-lower limb and had trouble walking. US showed an embolism in the left popliteal artery (Fig. 5), and contrast CT revealed that lung metastases were enlarged and the disease in the left-upper lobe had infiltrated the left superior pulmonary vein and left atrium (Fig. 6). Acute arterial occlusive disease in the left-lower limb caused by the tumor embolism was suspected, and she underwent endovascular thrombectomy. The tumor emboli were removed by embolectomy catheter and pathologically diagnosed as emboli from breast carcinoma with cartilaginous and osseous metaplasia (Fig. 7). After surgery, she underwent rehabilitation and regained her ability to walk. She then switched to domiciliary medical care at her request and passed away 3 months later.

## Discussion

Breast cancer with cartilaginous and/or osseous metaplasia is classified as a special type of invasive breast cancer that occurs in 0.003–0.12% of all cases [1, 2]. The clinical features include a larger tumor size than observed in other types of invasive ductal carcinoma and rapid growth. Lymph node metastasis has been reported in 23–35% of cases, which is a slightly lower percentage range than observed in other breast cancers. Conversely, hematogenous metastases early after surgery have been frequently observed, and the commonly reported metastatic sites are the lung, brain, and bone. As in our case, ER and PgR statuses are often negative, and chemotherapy resistance occurs in many cases [2]. In a previous report, patients with tumors that had pseudosarcomatous-predominant components were associated with worse prognosis than those with epithelial-predominant components (5-year overall survival: 28% vs. 62%) [2, 3].

Arterial tumor embolisms are rare. Although we searched the word “arterial tumor embolism” and “breast cancer” by PubMed, no cases of acute arterial tumor embolism associated with breast cancer have been reported. There are no reports that breast cancer with cartilaginous and/or osseous metaplasia is more prone to tumor embolism than other histologic types. Arterial tumor embolisms are often associated with primary or metastatic pulmonary malignancies that have invaded the veins or the left atrium. Surgical manipulation, tumor size, and growth rate have been cited as the main causes of arterial tumor embolisms. The recommended treatment is heparinization and embolectomy using a catheter [4]. Invasions of primary pulmonary malignancies into the pulmonary veins and extension into the left atrium are not uncommon. These invasions frequently occur in non-small cell carcinomas, such as squamous cell carcinoma, adenocarcinoma, and large-cell carcinoma among primary lung cancers. Additionally, this invasion type has also been reported in primary pulmonary sarcomas and malignant epithelioid leiomyoblastoma of the lung. On the other hand, invasion of metastatic lung tumors into the pulmonary veins and extension into the left atrium are very rare [5]. However, cases due to lung metastases from uterine carcinoma (choriocarcinoma and cervical carcinoma) and chondrosarcoma have been reported [6–9]. Cases in which the disease was surgically resected and those in which surgery was not thought to be indicated because of the disease extent have been reported [10]. Because the patient strongly desired to maintain quality of life and declined aggressive treatment, no treatment for her left-atrium thrombus was performed. If aggressive treatment had been desired, local treatment may have been the only option because breast cancer with cartilaginous and/or osseous metaplasia is not expected to respond to chemotherapy.

## Conclusion

It is hoped that this report of lung metastasis from breast cancer with cartilaginous and/or osseous metaplasia with acute lower-limb artery occlusion due to a tumor thrombus will be a useful addition to the literature because of the extreme rarity of such cases.

## Data Availability

The authors declare that all the data in this article are available within the article.

## Abbreviations

NAC: Neoadjuvant chemotherapy
US: Ultrasonography
CT: Computed tomography
ER: Estrogen receptor
PgR: Progesterone receptor
HER2: Human epidermal growth factor receptor 2
CT: Computed tomography
CEF: Cyclophosphamide, epirubicin, and fluorouracil
SLNB: Sentinel lymph node biopsy
